# Anaplastic Lymphoma Kinase (ALK)-Positive Anaplastic Large Cell Lymphoma Presenting as an Axillary Breast Abscess in a Young Woman: A Case Report

**DOI:** 10.7759/cureus.99911

**Published:** 2025-12-23

**Authors:** Dennis Amamnkwah, Firas Ibrahim

**Affiliations:** 1 Breast Surgery, Swansea Bay University Health Board, Swansea, GBR

**Keywords:** axillary abscess, cd30+ anaplastic large cell lymphoma, diagnostic delay, soft tissue tumours, young woman

## Abstract

Malignant tumours - both lymphoid and epithelial - have been reported to present clinically and radiologically as abscesses, leading to significant diagnostic delays. Anaplastic large cell lymphoma (ALCL) is a rare subtype of T cell non-Hodgkin lymphoma (NHL) characterised by CD30 positivity and, in some cases, anaplastic lymphoma kinase (ALK) expression. Although nodal involvement is most common, extranodal or soft-tissue disease may present with abscess-like features, resulting in diagnostic delay. A 31-year-old woman presented with right axillary pain and swelling, initially diagnosed as an abscess. Despite multiple courses of antibiotics and repeat imaging, the lesion enlarged and became indurated. An initial biopsy showed only chronic inflammation. Surgical incision and drainage were later performed, and histopathological analysis of tissue fragments revealed atypical lymphoid proliferation. Immunohistochemistry confirmed CD30-positive, ALK-positive ALCL.

Staging CT demonstrated a necrotic right axillary mass measuring 6 × 5.6 × 5.4 cm with splenomegaly (15 cm), but no mediastinal, retroperitoneal, or pelvic disease, as such findings were consistent with stage III(S) disease. The patient received brentuximab vedotin plus cyclophosphamide, doxorubicin and prednisone (BV-CHP) chemotherapy following multidisciplinary review and showed marked improvement. This case illustrates the diagnostic challenge of ALK-positive ALCL presenting as a recurrent axillary abscess. Early recognition and histopathological evaluation of atypical or non-resolving abscesses are essential for timely diagnosis and effective treatment, ultimately improving patient outcomes.

## Introduction

Soft-tissue tumours can sometimes masquerade as abscesses, leading to challenges in diagnosis. Several reported examples illustrate the importance of maintaining a high index of suspicion and performing histopathological evaluation in so-called abscesses that are atypical, recurrent or refractory to standard therapy.

In the breast and axilla, neoplastic processes may also present as such. Primary non-Hodgkin lymphoma (NHL) of the breast presenting as a breast abscess has previously been reported in the literature [1]. Although abscesses are typically benign infectious processes, in rare cases, they can mask underlying neoplasms.

We report the case of a 31-year-old woman who was referred to the breast surgery team with a presumed right axillary abscess. The patient initially developed pain in the right axilla, followed by the appearance of a lump, which was clinically and radiologically suspected to represent an abscess. Initial serial imaging and biopsy did not reveal any malignancy. Persistent symptoms and enlargement of the lesion despite multiple courses of antibiotics led to incision and drainage, during which further sampling from the abscess cavity revealed CD30-positive, anaplastic large cell lymphoma (ALK-positive ALCL).

Clinical and imaging findings were consistent with stage III(S) disease. The patient demonstrated excellent clinical response with progressive resolution of the axillary lesion following wound care, a short course of corticosteroids and initiation of chemotherapy. This case highlights a rare presentation of ALK-positive ALCL masquerading as a recurrent axillary abscess, emphasising the diagnostic challenges of lymphoma presenting as an apparent soft-tissue infection.

## Case presentation

**Figure 1:**
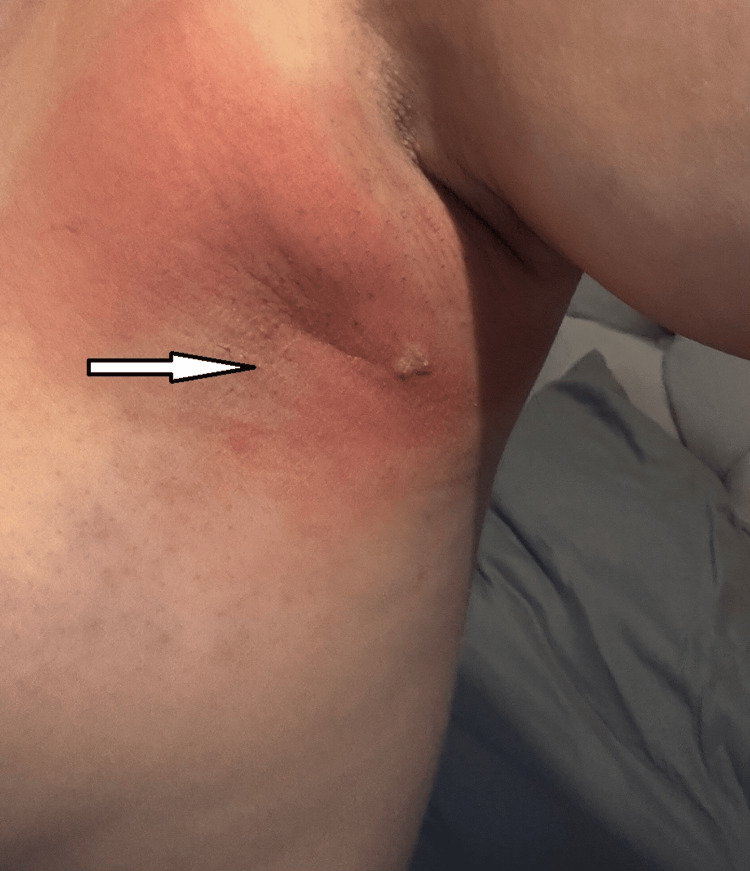
Initial presentation of a tender, erythematous swelling overlying the axillary tail (arrow).

A 31-year-old woman presented with a five-day history of right-sided chest pain. She denied any cough, hemoptysis, dyspnea or recent infection. Her past medical history included well-controlled asthma, mitral valve prolapse and a vasovagal episode. She was a non-smoker, and her maternal grandmother had breast cancer. On examination, the chest was clear with no tenderness or focal findings. An ECG showed normal sinus rhythm, and a chest X-ray demonstrated mild bronchovascular markings in the right lower zone but no consolidation or suspicious lesions. A diagnosis of musculoskeletal chest pain was made, and she was discharged with analgesia. Three days later, she re-presented with central chest pain occurring intermittently, mainly on exertion, and associated with mild shortness of breath. Further cardiac and respiratory assessment did not reveal any abnormality. Seven weeks later, the patient developed right axillary pain and swelling. On examination, there was a 3 x 2 cm soft tissue swelling in the anterior right axilla without skin changes or fluctuation (Figure 1).

**Figure 2:**
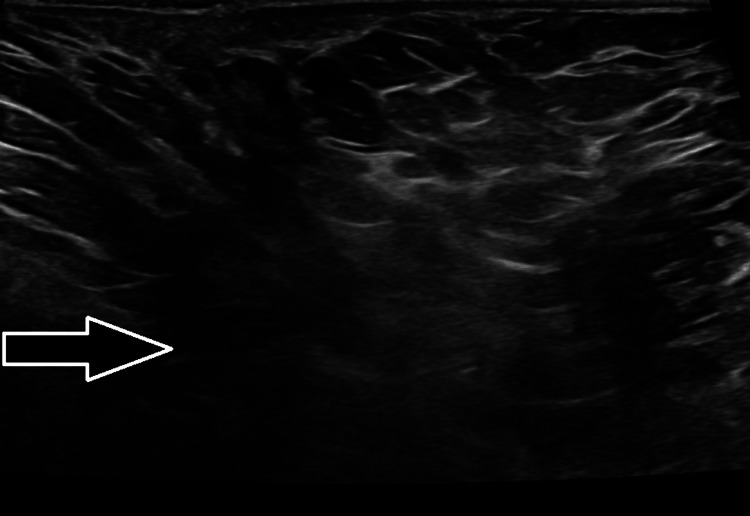
Ultrasound scan at initial presentation (arrow).

Ultrasound of the right axilla showed a small 3 × 2 cm hypoechoic collection with adjacent inflamed subcutaneous fat. This was located approximately 2 cm beneath the skin, with no significant lymphadenopathy. The left axilla was normal. The differential diagnosis included a small subcutaneous axillary abscess; however, due to its inferior location, a breast tail abscess could not be excluded, although it was considered less likely (Figure 2).

The patient was treated with a one-week course of oral flucloxacillin (500 mg, four times daily) as first-line therapy, given that she had only a small abscess, with a repeat ultrasound planned in one month to assess resolution. A week later, she re-presented with progression of the right axillary abscess, which was now slightly larger, tender, and extending medially into the breast and laterally approximately 4 cm beyond the axillary crease, with no surrounding cellulitis.

**Figure 3:**
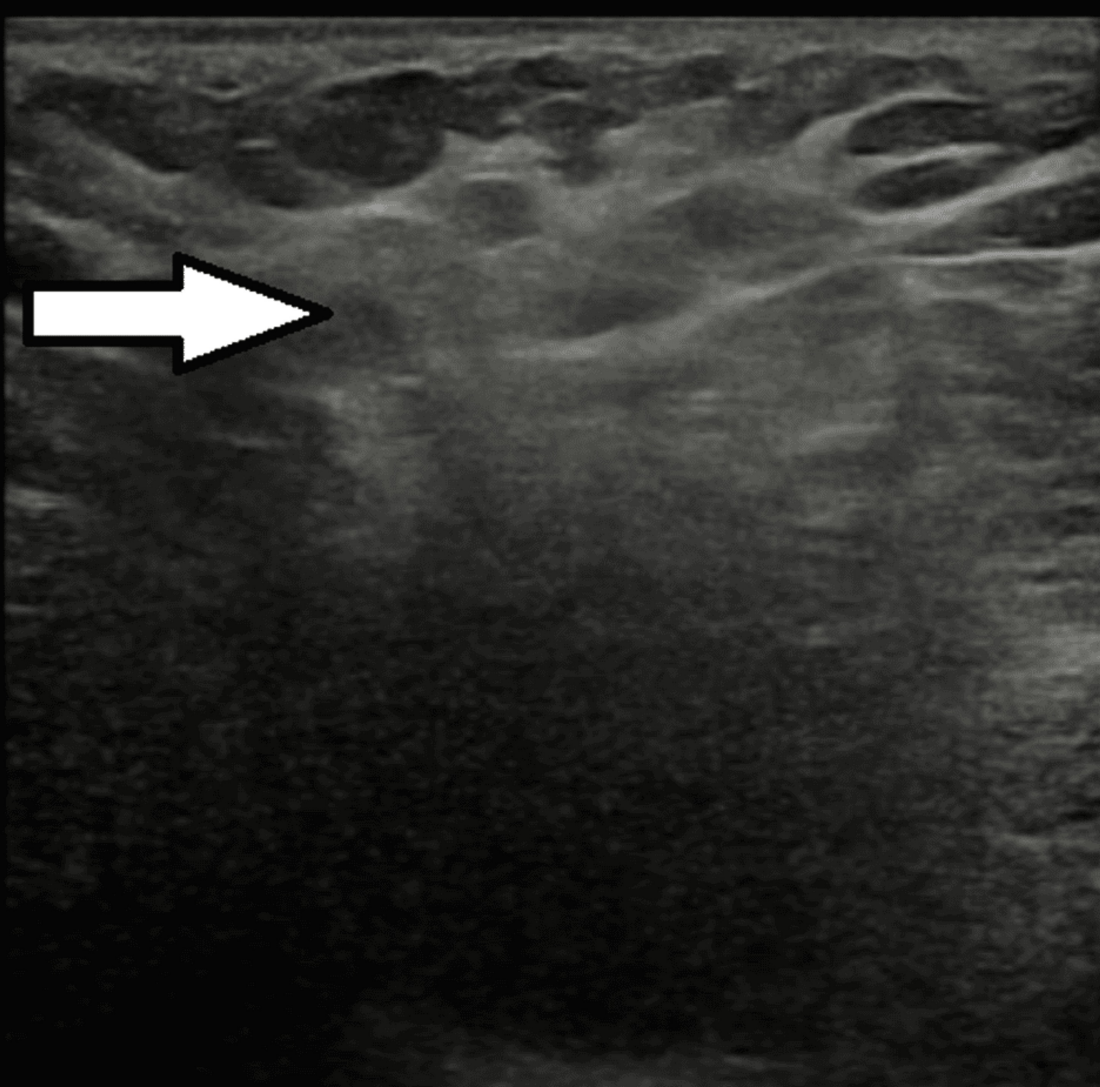
Repeat ultrasound after one week demonstrating a deep-seated abscess (arrow).

Repeat ultrasound, compared with the previous study, demonstrated an ill-defined hypoechoic area within the right axilla measuring 38 × 24 mm, with no internal vascularity. The overlying subcutaneous tissue and skin appeared thickened. These findings were consistent with an abscess; however, the appearance was unusual (Figure 3).

On account of these findings, a precautionary biopsy of the right axilla revealed fat necrosis and marked chronic inflammation with no malignancy noted.

However, the patient was not progressing, with persistent inflammation and redness of the skin, and a further one-week course of oral flucloxacillin (500 mg, four times daily) was extended. Subsequent sonographic assessment showed an increase in the size of the deeply seated abscess from 32 mm to 38 mm. No definite fluid collection was demonstrated, and there was no indication for ultrasound-guided aspiration.

Her symptoms persisted, and the lesion continued to enlarge. Due to the size and location of the collection overlying the axillary breast tail, a large incision for drainage would have likely been cosmetically suboptimal. In addition, on account of a high likelihood of breast tissue involvement, a decision was made that drainage under the breast team was the best care and the patient's antibiotics were reviewed and switched to a week's course of oral doxycycline (100 mg, twice daily).

Surgical drainage was performed 16 days after the patient first presented with the axillary abscess. Intraoperatively, a large 8-10 cm cavity was identified with minimal drainage, and an abscess cavity shave was taken. She was advised to continue oral antibiotics. Histopathological examination of the curetted tissue revealed lymphoid tissue with marked atypia. Cytokeratin immunostaining was negative, excluding epithelial malignancy, and the specimen was referred to the lymphoma panel. Postoperatively, the wound was regularly irrigated with local anaesthesia and normal saline. The wound bed initially appeared clean and healthy.

A fortnight after abscess drainage, the wound began leaking haemoserous fluid. Wound culture and sensitivity testing showed no growth. A vacuum-assisted closure (VAC) dressing was applied to the wound. On subsequent review, the VAC dressing canister was noted to be full, and the wound had developed an open cavity with significant induration and firm tissue. Wound bag dressing was applied to the area to allow lymph fluid to drain, and the patient was referred to the wound care team. She had at least four courses of antibiotics over this period.

**Figure 4:**
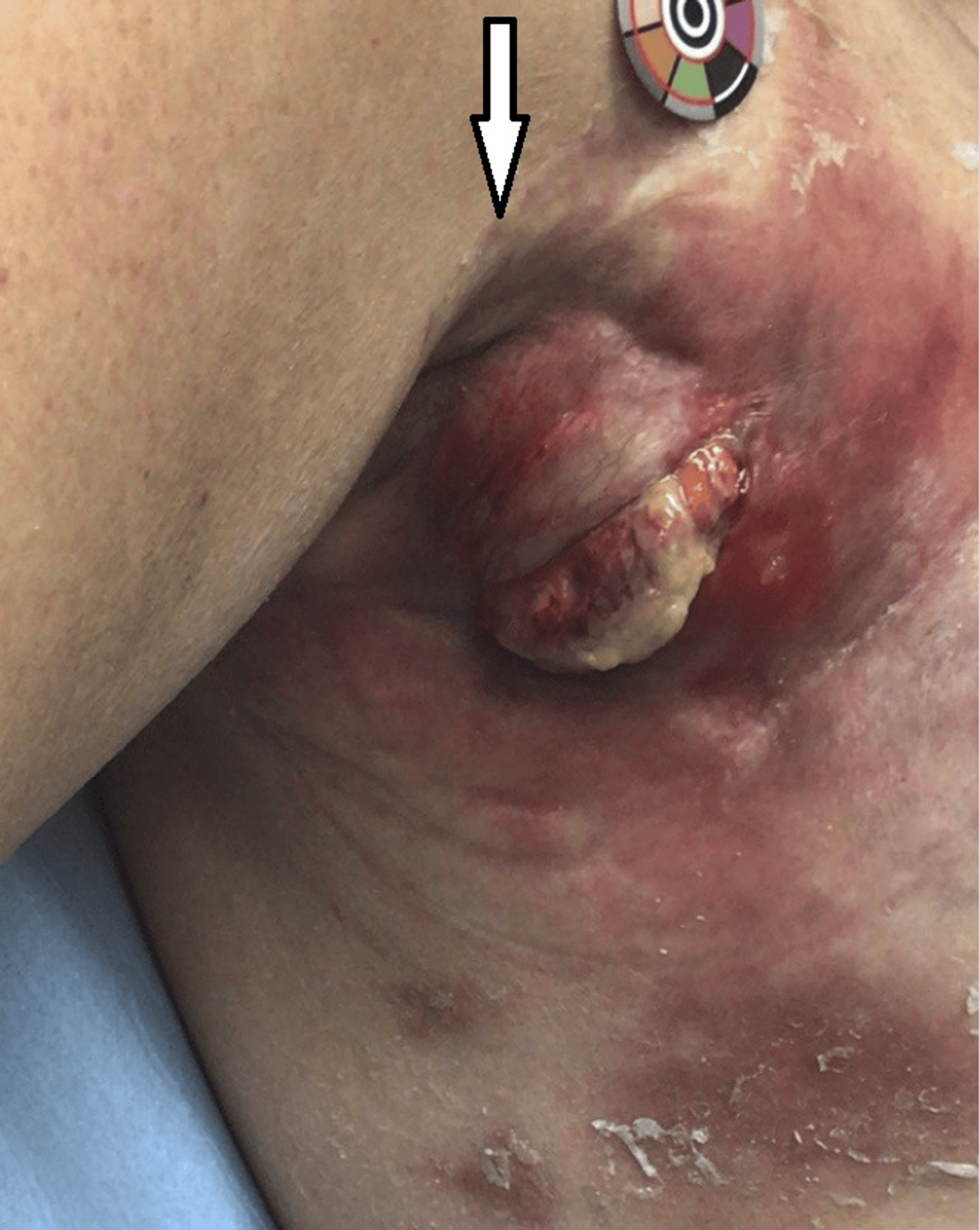
Fungating tissue arising from the wound (arrow).

Twenty-five days after the abscess drainage, the wound progressively dehisced, with the indurated firm tissue now fungating through the skin (Figure 4). There was no enlarged liver or spleen, and the abdomen was soft and non-tender. Systemically, she was well and reported no drenching night sweats, fevers or unexplained weight loss.

An integrated pathology report from the lymphoma panel concluded that the findings were consistent with ALK1-positive ALCL.

Sections showed an apparent lymph node effaced by a dense blastic lymphoid infiltrate with areas of karyorrhexis and apoptotic cells. There was conspicuous mitotic activity, but no Reed-Sternberg cells were identified, and there was no evidence of epithelial malignancy.

Immunophenotyping

**Figure 5:**
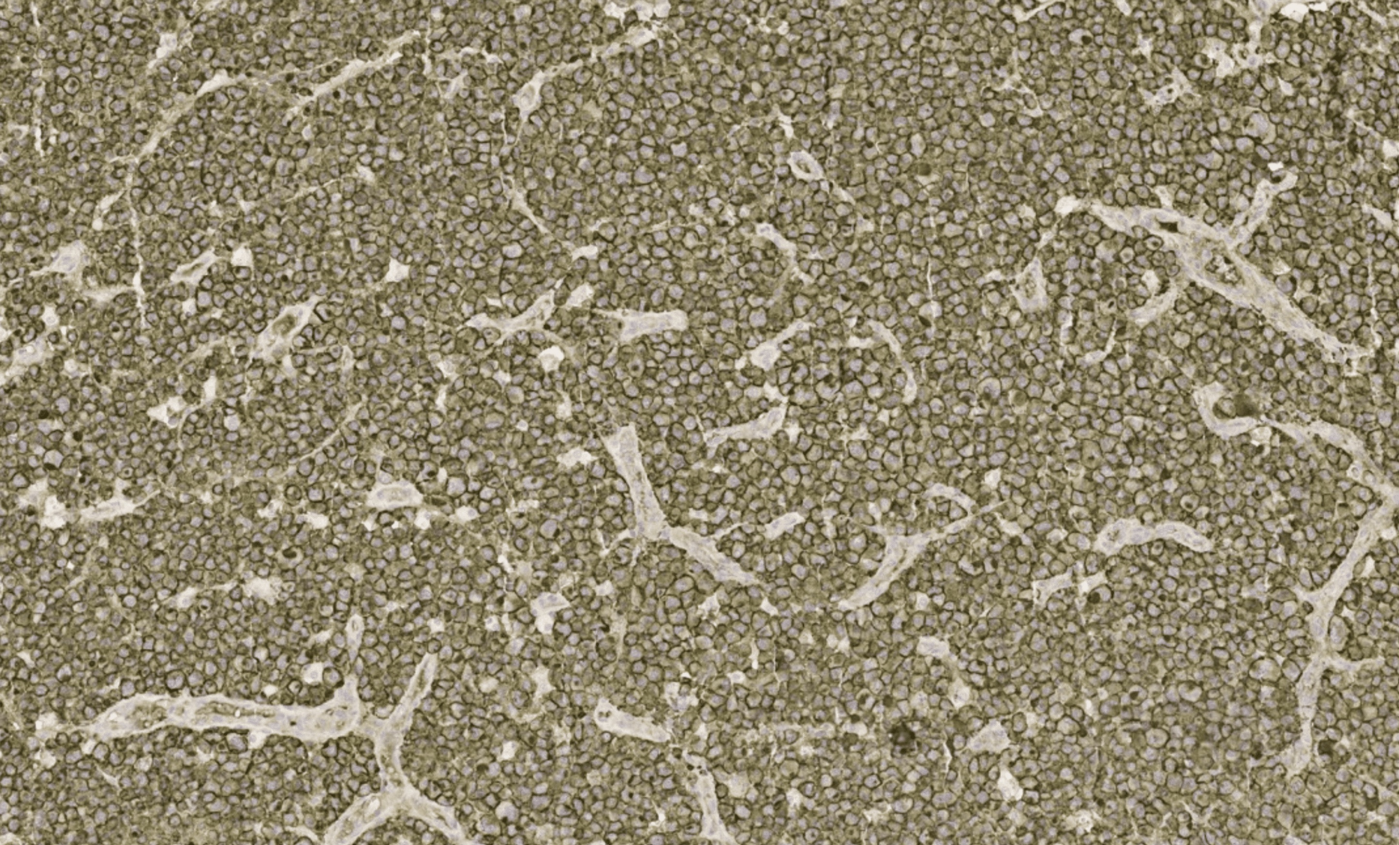
CD30 immunohistochemistry (IHC), ×10 magnification.

**Figure 6:**
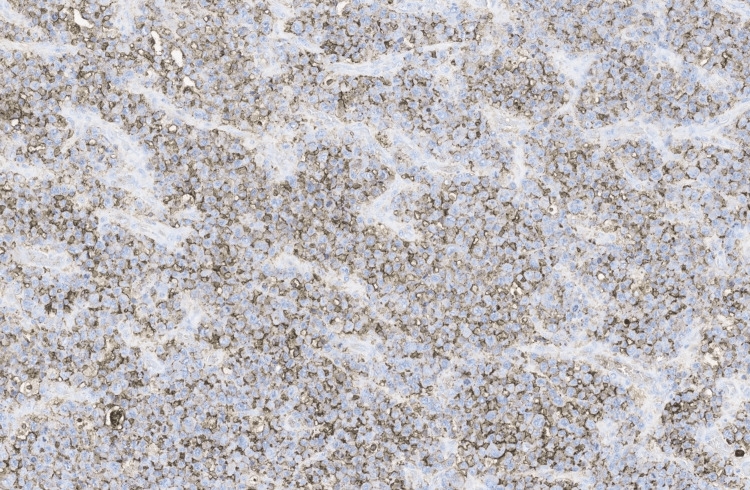
Epithelial membrane antigen (EMA) immunohistochemistry (IHC), ×10 magnification.

**Figure 7:**
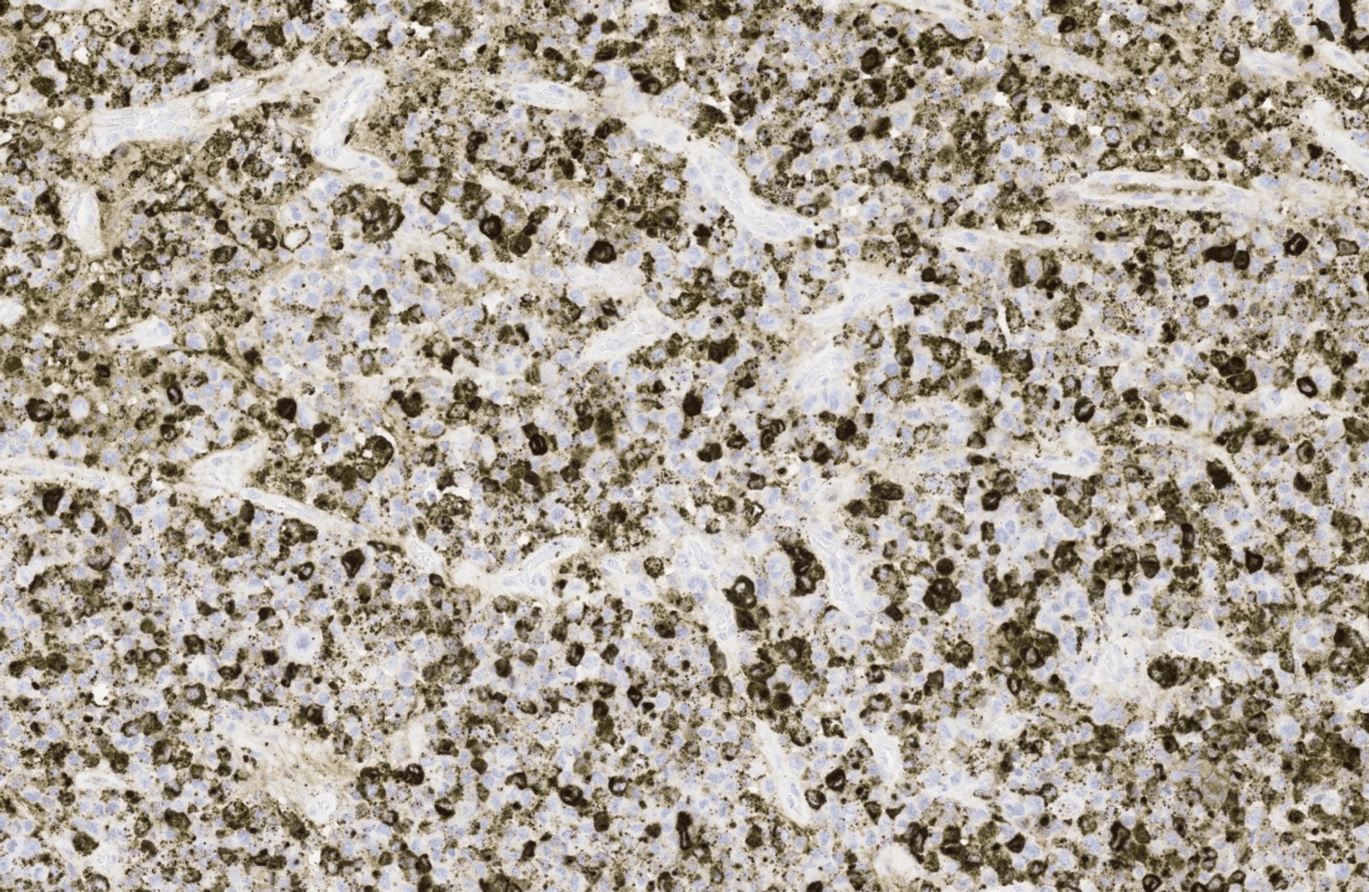
Granzyme B immunohistochemistry (IHC), ×10 magnification.

The lesional cells had a phenotype characterised by a strong cluster of differentiation (CD)30 positivity (Figure 5), ALK1+, multiple myeloma oncogene 1 (MUM1)+, and epithelial membrane antigen (EMA)+ (Figure 6). The cells showed CD45+/-, and were negative for CD3, CD7, CD2, CD5, CD43, CD79a, CD138, GATA3, PAX5, CD20, CD10, B-cell lymphoma 6 (BCL6), CD21, CD23, OCT2, and pan-cytokeratin. They were positive for granzyme B (Figure 7), perforin, T cell-associated intracellular antigen 1 (TIA-1), and CD8. c-MYC expression was negative. Genetic and molecular investigations showed no signal on Epstein-Barr virus-encoded RNA (EBER).

**Figure 8:**
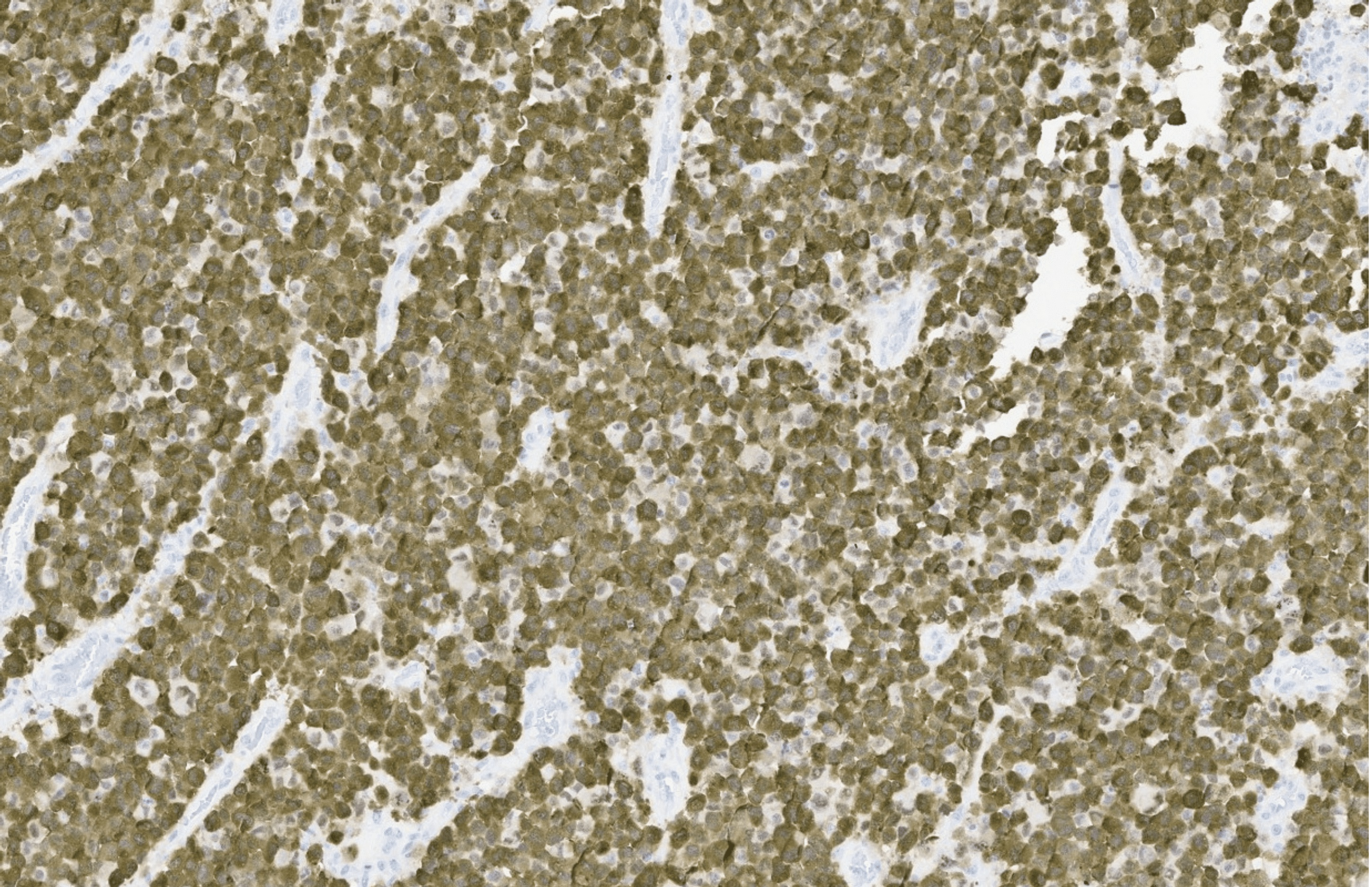
ALK1 immunohistochemistry (IHC), ×10 magnification.

**Figure 9:**
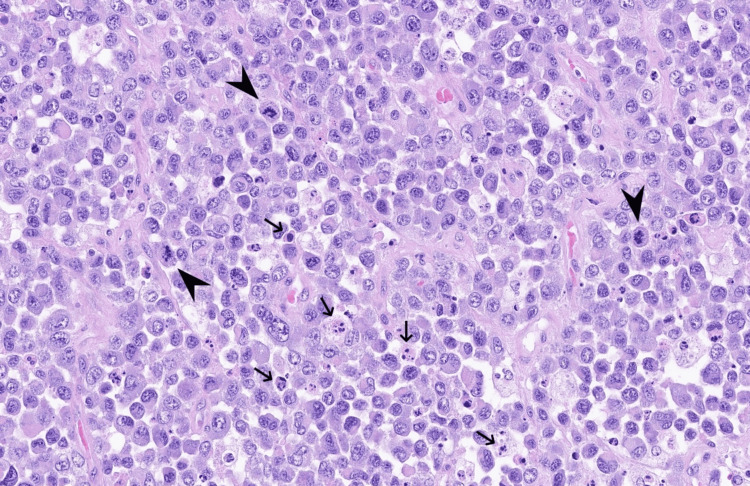
Haematoxylin and eosin (H&E) stain at ×20 magnification. The arrows indicate the atypical lymphoid cells, while the arrowheads highlight the large anaplastic cells with kidney- or horseshoe-shaped nuclei.

In conclusion, the morphological and immunophenotypic features were consistent with ALK1-positive ALCL (Figures 8-9).

A supplementary report revealed the following findings: “Karyotype: evidence of an ALK gene rearrangement. No evidence of an IRF4 (DUSP22) gene rearrangement. TP63 analysis failed.” Analysis of tissue sections demonstrated an abnormal signal pattern consistent with the presence of an ALK gene rearrangement, with no evidence of an IRF4 (DUSP22) gene rearrangement. However, an additional copy of the IRF4 (DUSP22) gene probe was identified. In conclusion, the presence of an ALK1 gene rearrangement supported the above diagnosis.

A nuclear medicine (NM) whole-body fluorodeoxyglucose positron emission tomography-computed tomography (FDG PET-CT) was suggested following the histology report and demonstrated an extremely FDG-avid infiltrating right axillary mass complicated by a draining abscess. There was a moderate generalised increase in FDG uptake throughout an expanded bone marrow, which was considered a reactive pattern. Given the prominent bone marrow uptake, the subtle generalised increase in FDG uptake within the spleen was also considered most likely reactive. Overall findings were suggestive of probable stage I disease, although a generalised marrow abnormality could not be excluded.

On account of the above findings, the patient was referred to the haematology multidisciplinary team (MDT) six weeks after the initial presentation as an abscess. The MDT staged the lymphoma as Ann Arbor stage III(S) and decided to initiate treatment with brentuximab vedotin plus cyclophosphamide, doxorubicin and prednisone (BV-CHP), then rediscuss for radiotherapy at a later stage. Wound care was continued alongside a short course of steroids, and the patient was also commenced on chemotherapy 10 weeks after initially presenting as an abscess. The lesion subsequently detached and was observed in the wound bag, and excellent granulation tissue was noted on follow-up review. Exudate had also decreased, and there was no odour. The spreading erythema had reduced, although the surrounding skin remained red.

**Figure 10:**
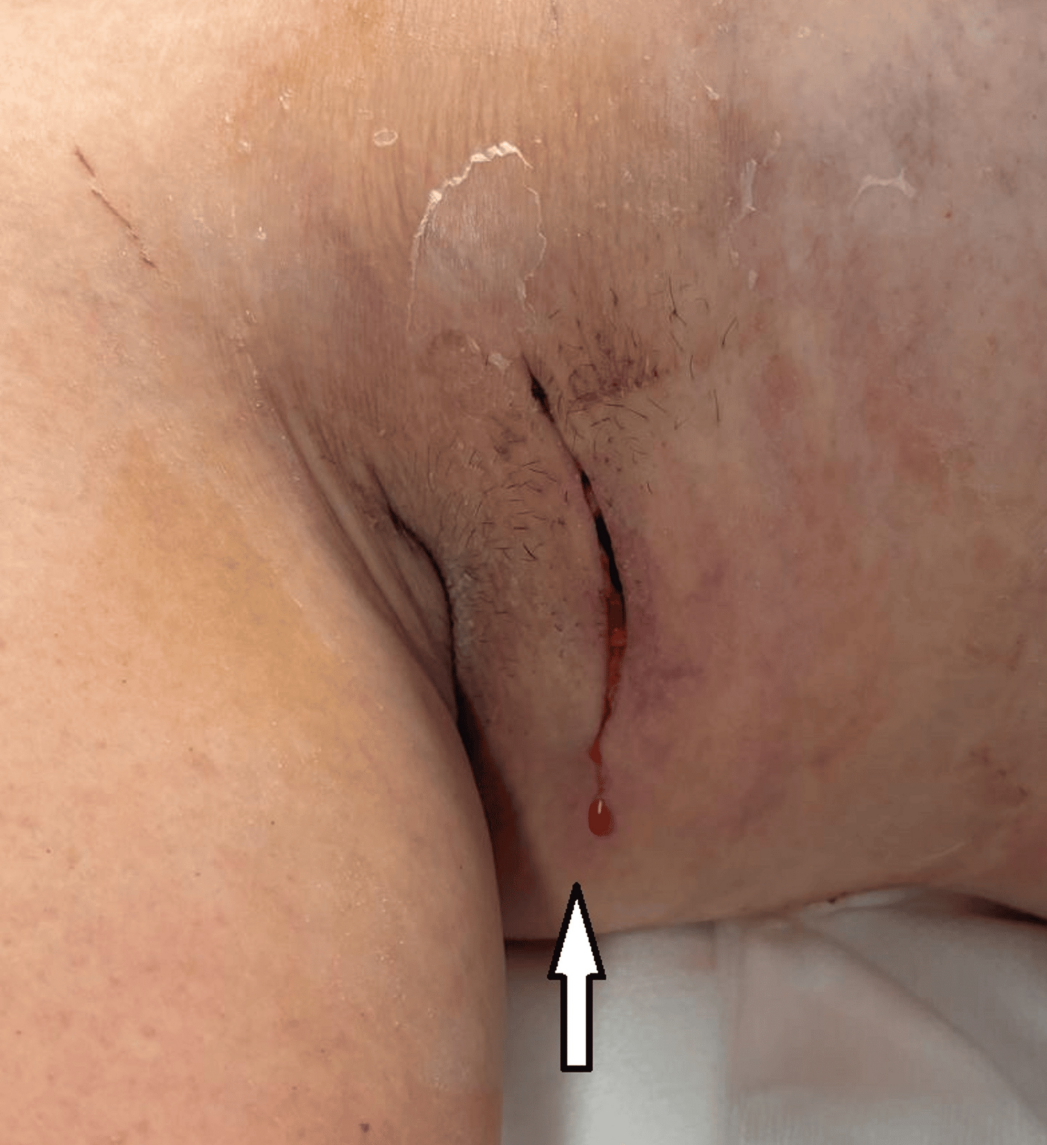
Healed wound following treatment (arrow).

The patient’s wound is now healed and dry 14 weeks after the initial presentation with an abscess, and she is currently doing well clinically under joint follow-up with the haematology and breast surgery teams (Figure 10).

## Discussion

ALCL is a distinct subtype of T cell NHL, first recognised by Stein et al. (1985) and later classified by the WHO as a CD30-positive T cell neoplasm characterised by large anaplastic lymphoid cells with pleomorphic nuclei and strong CD30 (Ki-1) expression [2]. ALCL accounts for approximately 2-3% of all NHLs and 12% of peripheral T cell lymphomas [3].

Based on ALK expression and clinical context, ALCL is divided into four main entities: systemic ALK-positive ALCL, systemic ALK-negative ALCL, primary cutaneous ALCL (pcALCL) and breast implant-associated ALCL (BIA-ALCL). ALK-positive ALCL arises from genetic rearrangements involving the ALK gene located on chromosome 2p23, most commonly the t(2;5)(p23;q35) translocation, which fuses ALK with the nucleophosmin (NPM) gene. This produces the NPM-ALK fusion protein, a constitutively active tyrosine kinase that drives oncogenesis through activation of multiple downstream signalling pathways (JAK/STAT3, PI3K/AKT and RAS/ERK), leading to uncontrolled cellular proliferation and resistance to apoptosis [4,5].

Histopathologically, ALK-positive ALCL demonstrates a diffuse proliferation of hallmark cells, i.e., large pleomorphic lymphoid cells with eccentric, horseshoe- or kidney-shaped nuclei and abundant cytoplasm. Immunophenotypically, these cells express CD30 and ALK, which are crucial for diagnosis and understanding the pathology of ALCL. The disease often presents with nodal involvement (cervical, axillary or inguinal), but extranodal disease, including bone, skin, soft tissue, liver and lung, occurs in 30-50% of cases [6]. B symptoms (fever, weight loss, night sweats) are present in roughly half of patients, though they were absent in our patient.

ALCL can occasionally present as a soft-tissue lesion that mimics an abscess, resulting in diagnostic delay. In this case, the patient’s right axillary lesion demonstrated necrosis, induration and discharge, features typical of bacterial infection, which led to multiple courses of antibiotics and surgical intervention before a definitive diagnosis was established. This clinical mimicry is well documented in the literature. Lozano-Jaramillo et al. (2023) described a soft-tissue pelvic ALK-positive ALCL initially treated as an inflammatory infection [6], and Nagasaka et al. (2000) reported cases of ALCL presenting primarily in bone or soft tissue, mimicking sarcoma or osteomyelitis [7].

This case highlights a rare clinical presentation of ALK-positive ALCL presenting as a recurrent axillary abscess, emphasising the need for early biopsy and histopathological evaluation of non-resolving or atypical soft-tissue lesions. Our patient, a 31-year-old woman, initially presented with a painful axillary swelling that was clinically and radiologically suggestive of an abscess. Despite multiple antibiotic courses and repeated ultrasound assessments, the lesion failed to resolve and progressively enlarged. Histopathological evaluation following incision and drainage ultimately revealed CD30-positive, ALK-positive T cell ALCL. Soft-tissue involvement in ALCL is uncommon but well documented, particularly ALK-positive ALCL cases in younger patients. Additionally, CD30 is commonly expressed in benign dermatological conditions, warranting close clinical consideration and follow-up [8]. The neoplasm often manifests as a rapidly enlarging, painful soft-tissue mass that may be necrotic or ulcerated, mimicking infectious or inflammatory lesions such as bacterial abscesses and leading to delays in oncologic referral and definitive management. The initial biopsy demonstrated chronic inflammation and fat necrosis - a common finding in abscess-like presentations of lymphoma [9].

The lesion’s persistence and enlargement despite antibiotics warranted further sampling, which proved crucial in reaching the correct diagnosis. This underscores that failure to respond to standard antimicrobial therapy should prompt reconsideration of the diagnosis and tissue sampling for histopathological evaluation. Histologically, ALCL is characterised by large pleomorphic cells with strong CD30 expression and a T/null-cell immunophenotype, with ALK expression conferring a favourable prognosis. In this case, the tumour demonstrated ALK1 positivity with the absence of DUSP 22 or TP63 rearrangements, supporting the diagnosis of systemic ALK-positive ALCL. The patient’s presentation without B symptoms and localised disease to the axilla and spleen suggested stage III(S) disease, aligning with prior reports describing extra-nodal soft-tissue involvement as a rare but recognised manifestation [6,7].

Key diagnostic immunophenotypic features (CD30+, ALK+, absence of B cell markers such as CD20 and PAX 5) confirmed the diagnosis. Such cases, including ours, highlight the need for early biopsy of atypical soft-tissue masses, particularly when there is a lack of clinical improvement after antibiotics, imaging shows non-specific hypoechoic or necrotic lesions without clear abscess formation, or there is progressive induration or ulceration.

Following diagnosis, our patient was commenced on BV-CHP, which represents the current standard of care for CD30-positive peripheral T cell lymphomas. The ECHELON-2 phase III trial demonstrated that BV-CHP significantly improved progression-free and overall survival compared with cyclophosphamide, doxorubicin, vincristine and prednisone (CHOP) [10]. The patient showed an excellent early response, with lesion regression and improved wound healing following combined wound management, a short course of steroids and initiation of chemotherapy. Given her ALK positivity and limited-stage disease, the prognosis is favourable, though long-term follow-up is essential due to the risk of relapse or secondary cutaneous involvement.

## Conclusions

This case underscores the diagnostic challenges of ALK-positive ALCL presenting as a recurrent axillary abscess. The patient’s initial clinical and radiological findings were indistinguishable from an infectious process, resulting in multiple courses of antibiotics. Lack of response to multiple courses of appropriate antibiotics, progressive enlargement and induration despite surgical treatment, unusual sonographic appearances and minimal drainage upon incision ultimately prompted repeat surgical exploration and histopathological reassessment, which revealed CD30 and ALK positivity consistent with systemic ALCL. This highlights the need to maintain a high index of suspicion for malignancy in atypical or non-resolving soft-tissue abscesses and to pursue early biopsy and multidisciplinary evaluation when conventional management fails.

The patient demonstrated an excellent response to BV-CHP, with complete wound healing and remission, illustrating the effectiveness of targeted therapy in ALK-positive disease. This case emphasises that prompt recognition and accurate histopathological diagnosis are critical to avoid treatment delays and improve outcomes. Clinicians should consider lymphoma in the differential diagnosis of recurrent or refractory abscess-like lesions, particularly in young patients, to enable early intervention and optimise prognosis.
